# Electrochemical Sensor Based on DNA Aptamers Immobilized on V_2_O_5_/rGO Nanocomposite for the Sensitive Detection of Hg(II)

**DOI:** 10.3390/s25072334

**Published:** 2025-04-07

**Authors:** Mahesh A. Takte, Shubham S. Patil, Akash V. Fulari, Tibor Hianik, Mahendra D. Shirsat

**Affiliations:** 1 RUSA Center for Advanced Sensor Technology, Department of Physics, Dr. Babasaheb Ambedkar Marathwada University, Chhatrapati Sambhajinagar 431004, MS, India; mahesh05takte@gmail.com (M.A.T.); shubhamspatil10297@gmail.com (S.S.P.); 2 Symbiosis Centre for Nanoscience and Nanotechnology, Symbiosis International (Deemed University), Pune 412115, MS, India; akashfulari@gmail.com; 3 Department of Nuclear Physics and Biophysics, Faculty of Mathematics, Physics and Informatics, Comenius University, Mlynska dolina F1, 84248 Bratislava, Slovakia; 4 Department of Electronics, Dr. Babasaheb Ambedkar Marathwada University, Chhatrapati Sambhajinagar 431004, MS, India

**Keywords:** electrochemical sensor, Hg(II), DNA aptamers, V_2_O_5_/rGO nanocomposite, differential pulse voltammetry

## Abstract

**Highlights:**

**What are the main findings?**

- A highly sensitive electrochemical aptasensor based on a V_2_O_5_/rGO nanocomposite was developed.
- A detailed characterization of the nanocomposite materials was performed by advanced physical methods.

**What is the implication of the main finding?**

- The aptasensor allowed for the sensitive and selective detection of Hg(II) below the maximum permissible limit of Hg(II).
- The sensor revealed a stable response for 40 days.

**Abstract:**

We developed a sensor consisting of V_2_O_5_ nanorods and a reduced graphene oxide (rGO) nanocomposite (V_2_O_5_/rGO) with immobilized DNA aptamers (Apt-NH@V_2_O_5_/rGO) for the sensitive electrochemical detection of Hg (II). The V_2_O_5_ nanorods anchored on rGO nanosheets were synthesized using a hydrothermal method. The nanocomposite was analyzed by various powerful physical methods that include X-ray diffraction (XRD), energy-dispersive X-ray spectroscopy (EDX), field emission scanning electron microscopy (FE-SEM), Raman spectroscopy, the Brunauer–Emmett–Teller (BET) method, and Fourier transform infrared spectroscopy (FTIR). The FE-SEM of V_2_O_5_ disclosed the nanorod-like structure and uniform anchoring of V_2_O_5_ on the rGO nanosheet. Moreover, the BET results showed that the V_2_O_5_/rGO nanocomposite possesses excellent porosity. Furthermore, a glassy carbon electrode (GCE) was modified with Apt-NH@V_2_O_5_/rGO and used for the electrochemical detection of Hg(II) by differential pulse voltammetry (DPV). The aptasensor exhibited excellent sensitivity and selectivity toward Hg(II) detection, with a limit of detection (LOD) of 5.57 nM, which is below the maximum permissible limit established by WHO for rivers (30 nM). The sensor also exhibited significant stability and good repeatability.

## 1. Introduction

The pollution of aquatic ecosystems by heavy metal ions (HMIs) is a serious concern due to their harmful effect on marine life, as well as on the lives of hundreds of millions of individuals worldwide [1,2]. Among the HMIs, mercury (Hg(II)) is the most dangerous to human health [3,4,5] due to its high reactivity and accumulation effect. Moreover, a low-level dose of Hg(II) can cause serious issues in the central nervous system and harm the brain, lungs, kidneys, and the growing fetus. Therefore, there is an urgent need for the selective and sensitive detection of Hg(II) contamination. Generally, cold vapor atomic absorption spectroscopy, atomic fluorescence spectroscopy, and inductively coupled plasma mass spectrometry (ICP-MS) have been utilized for the detection of Hg(II) [6,7,8]. These methods are precise and accurate for the detection of HMIs, but they rely on sophisticated, expensive instrumentation and have high operating costs, which are not suitable for the rapid monitoring of samples in the field. In contrast, electrochemical techniques typically do not require such expensive instrumentation and are suitable for the on-site and routine detection of Hg(II) [9] with high selectivity and sensitivity. Among the various electrochemical methods, differential pulse voltammetry (DPV) shows high sensitivity and the potential for detecting Hg(II) in its natural environment [10].

To date, many researchers have worked on the electrochemical detection of Hg(II) using different materials. For example, Erçarıkcı et al. [11] developed an rGO/ZnO-NPs-ED composite for the simultaneous electrochemical detection of Cd(II), Pb(II), Cu(II), and Hg(II), demonstrating effective Hg(II) adsorption. However, the selectivity for Hg(II) was limited, and the sensor detected mercury ions in a relatively narrow linear range (3.3–300 µM), with a limit of detection (LOD) of 1.0 µM. Moreover, Cheng et al. [12] synthesized a Co_3_O_4_/ZnO composite via a one-step hydrothermal method and modified a glassy carbon electrode (GCE) for Hg(II) detection using square-wave anodic stripping voltammetry (SWASV). While this sensor exhibited good stability, it demonstrated a relatively high LOD of 0.3 µM and poor selectivity. An overview of the existing literature highlights the necessity for the development of an Hg(II) sensor that exhibits improved sensing parameters, including high sensitivity, selectivity, stability, and repeatability. It is also evident that the physicochemical properties of nanomaterials are crucial for the improved electrochemical efficiency of the sensors [13].

In this regard, to enhance the electrochemical efficiency of sensors, various metal oxide nanomaterials have been utilized for the detection of HMIs [14,15]. Among these, the nanostructured transition metal oxide vanadium pentoxide (V_2_O_5_) is an N-type semiconductor with a direct optical band gap of 2.3 eV [16]. Meanwhile, V_2_O_5_ possesses excellent electrochemical properties, as well as a low cost, a layered structure, and extensive oxidation states (viz. V^2+^, V^3+^, V^4+^, and V^5+^) [17]. However, the typical conductivity of V_2_O_5_ is limited. Therefore, for the preparation of the optimal nanocomposite of V_2_O_5_, a support carrier with good conductivity has gained the focus of researchers. In this context, the incorporation of graphene derivatives, which have excellent properties, such as a high surface area and superb electron transport kinetics, is rather advantageous [18,19]. Specifically, reduced graphene oxide (rGO) is a potential candidate for nanocomposite preparation due to its excellent properties, such as high conductivity, insolubility in water, and the presence of an oxygen functional group on the surface, which has an affinity toward metal ions [20,21,22]. In addition, the composition of rGO with V_2_O_5_ will not only overcome the conductivity issue but also enhance the analyte’s adsorption ability, improving the electrochemical performance.

Moreover, the selectivity of the detection of various analytes can be provided by nucleic acid aptamers. DNA or RNA aptamers are short, single-stranded oligonucleotides composed typically of 15–80 bases that, in a solution, fold into a 3D structure, forming a binding site with the analyte for which they have been developed. DNA aptamers are preferable in comparison to RNA-based oligonucleotides due to their better stability and lower costs. The aptamers are also known as chemical antibodies. But, in contrast to antibodies, they are developed in vitro by a combinatorial chemistry method known as SELEX (Systematic Evolution of Ligands by EXponential enrichment). Once the aptamer sequence is determined, it can be reproduced with high precision. Moreover, the aptamers can be chemically modified on one or both sides, which increases their stability against cleavage by nucleases and allows for their covalent immobilization on various surfaces [23]. The aptamers present the benefits of specificity and cost-efficient development and exhibit stronger binding affinity compared to small-molecule ligands [24]. Therefore, the composition of aptamers with metal oxides through covalent bonding has numerous advantages in sensor applications over traditional methods. The utilization of nanomaterials based on metal oxides and rGO increases the surface area, which raises the density of the immobilized aptamers and, thus, improves the sensitivity of detection [25]. In general, Hg(II) detection by DNA aptamers is based on thymine (T) mismatches, which result in the formation of stable T − Hg^2+^ − T complexes [26]. This approach has been used in several recent papers for Hg(II) detection. For example, Ulloa-Gomez et al. [27] prepared a paper-based microfluidic (μ-PAD) aptasensor for the determination of Hg(II) with high selectivity and an LOD of 5 ppm (25 nM). Moreover, Su et al. [28] developed an aptasensor based on reduced graphene oxide (rGO) and gold nanoparticles (AuNPs) for the electrochemical detection of Hg(II). The aptasensor exhibited long-term stability, a wide dynamic range, and a good LOD of 1.2 µg/L (6 nM), but the selectivity was limited. Tian et al. [29] proposed an electrochemiluminescence (ECL)-based aptasensor using a functionalized rare-earth cerium oxide (CeO_2_) as the luminescent unit and an aptamer as a capture unit composed of several T mismatches. The formation of the T − Hg^2+^ − T bridges in the presence of Hg^2+^ resulted in changes in the ECL signal. The sensor revealed good selectivity and stability for 7 days, with a dynamic range of 10 pM–100 μM in a logarithmic scale and an LOD of 0.35 pM. The detection of Hg(II) in spiked fish and shrimp samples demonstrated relatively good recovery between 82.99 and 105%. An electrochemical aptasensor based on a black-phosphorus porous graphene nanocomposite was reported by Zhou et al. [30]. Using DPV, it was possible to detect Hg^2+^ ions with an LOD of 45 pM in a logarithmic linear range of 1 nM–10 µM. The sensor was validated in river samples with good recovery of 93.68–102.43%. More recently, Chen et al. [31] reported a field effect transistor (FET)-based aptasensor for the detection of Hg(II). They designed a temperature-stable hairpin structure that allowed for the formation of the T − Hg^2+^ − T bridges at low mercury concentrations with an LOD of 4.68 pM. For the optimized hairpin structure, the linear range in the logarithmic scale was 10^−11^–10^−5^ M of Hg(II). They obtained good correlation for the detection of Hg(II) in the Touqian River in Hsinchu (Taiwan) by the aptasensor (0.007 ppb) and by ICP-MS (0.012 ppb), which is below the maximum permissible limit (MPL) for mercury, established by the WHO for river water (6 ppb) [32].

In this work, we hydrothermally synthesized a nanocomposite of V_2_O_5_ and rGO (V_2_O_5_/rGO), which was employed to modify the surface of a GCE. The GCE modified by V_2_O_5_/rGO was functionalized with amino-modified aptamers (Apt-NH), forming a sensing surface of Apt-NH@V_2_O_5_/rGO, which provided the novel approach for the electrochemical sensing of Hg(II) ions with high sensitivity. The developed aptasensor showed excellent selectivity, repeatability, stability, and an LOD of 5.57 nM, which is below the MPL (6 ppb; 30 nM) for river water [32].

## 2. Materials and Methods

### 2.1. Chemicals

Vanadium (IV) oxide bis (2,4-pentanedionate) (C_10_H_14_O_5_V) was purchased from Alfa Aesar Chemicals, New York, USA. Vanadium pentoxide (bulk V_2_O_5_), ethylene glycol (C_2_H_6_O_2_), sulfuric acid (H_2_SO_4_), hydrochloric acid (HCl) of p.a. grade, and mercury chloride (HgCl_2_) were purchased from Molychem, Mumbai, India. Copper chloride (CuCl_2_.2H_2_O) and lead nitrate (PbNO_3_)_2_ were purchased from MI Modern Science Labs Pvt. Ltd., Nashik, Maharashtra, India. These metal salts were used for the preparation of the HMI solutions. Hydrogen peroxide (H_2_O_2_) was acquired from Modern Industries, Mumbai, India. Graphite fine powder was sourced from Loba Chemie PVT.LTD., Tapukara, Alwar, Rajasthan, India. Potassium permanganate (KMnO_4_) was acquired from HPLC PVT. LTD., Mumbai, India. 1-Ethyl-3-(3-dimethylaminopropyl) carbodiimide (EDC), N-hydroxysuccinimide (NHS), Tris-HCl, K_3_[Fe(CN)_6_], and K_4_[Fe(CN)_6_] were purchased from Merck (Darmstadt, Germany). Milli-Q (DI) with a resistivity of 18.0 MΩ.cm, prepared by Purelab Classic UV (Elga, High Wycombe, UK), was used for the preparation of the water solutions. All of the specified chemical reactants were used as received without any additional purification. A DNA aptamer, modified by an amino-linker (Apt-NH) with the composition of 5′-NH_2_-TTT TTT TTT GGG GGC ACA CAT GTA GGT GCT GTC CAG GTG TGG TTG TGG T-3′ was purchased from GeneCust (Boynes, France). This oligonucleotide has several T-T mismatches that can form complexes with Hg(II).

### 2.2. Preparation of V_2_O_5_/rGO

A nanorod-like V_2_O_5_ was prepared using a simple hydrothermal method. Specifically, 30 mL of DI and bulk V_2_O_5_ (3.6 mM) was stirred magnetically for 35 min to obtain a homogeneous dispersion. Subsequently, in the obtained dispersion, 22.5 mL of 30 wt% H_2_O_2_ was added, and the dispersion was ultrasonicated for 20 min. After that, the brownish-colored solution was enclosed in a 100 mL Teflon-lined stainless-steel autoclave and kept in an electric oven for 12 h at 180 °C. After the autoclave was naturally cooled, the solution was collected and filtered using ethanol and DI. Then, the resulting residue was dried overnight at 80 °C. The final product was obtained by annealing the sample at 200 °C.

The preparation of GO followed the enhanced Hummers’ method, as detailed in our previous publication [33]. For this purpose, the graphite powder (6.0 g) and NaNO_3_ (3.0 g) were dissolved in 138 mL of H_2_SO_4_, and the solution was magnetically stirred for 2 h. Afterwards, KMnO_4_ (18.0 g) was cautiously added to the solution, while the solution was kept in an ice bath with constant magnetic stirring. In the next step, the mixture was heated to 35 °C with continuous stirring in an oil bath for 30 min. Meanwhile, ice (276 mL) was gently added to the solution, which produced an exothermic reaction, and, thereafter, it was kept on the hotplate for 15 min at 95 °C. After the solution was naturally cooled, 3 mL of H_2_O_2_ and 420 mL of DI were added to the reaction to stop the oxidation process.

Afterwards, the obtained solution was filtered by the following process: Firstly, the solution was passed through a polyester fiber. Then, the filtrate was centrifuged for 2 h at 4000 RPM, and the collected precipitate was progressively washed with 30% HCl, ethanol, and DI. The final material was collected by drying the precipitate overnight at ambient conditions. Finally, the rGO was obtained by heating the GO at 350 °C until self-combustion after 15 min.

**Scheme 1:**
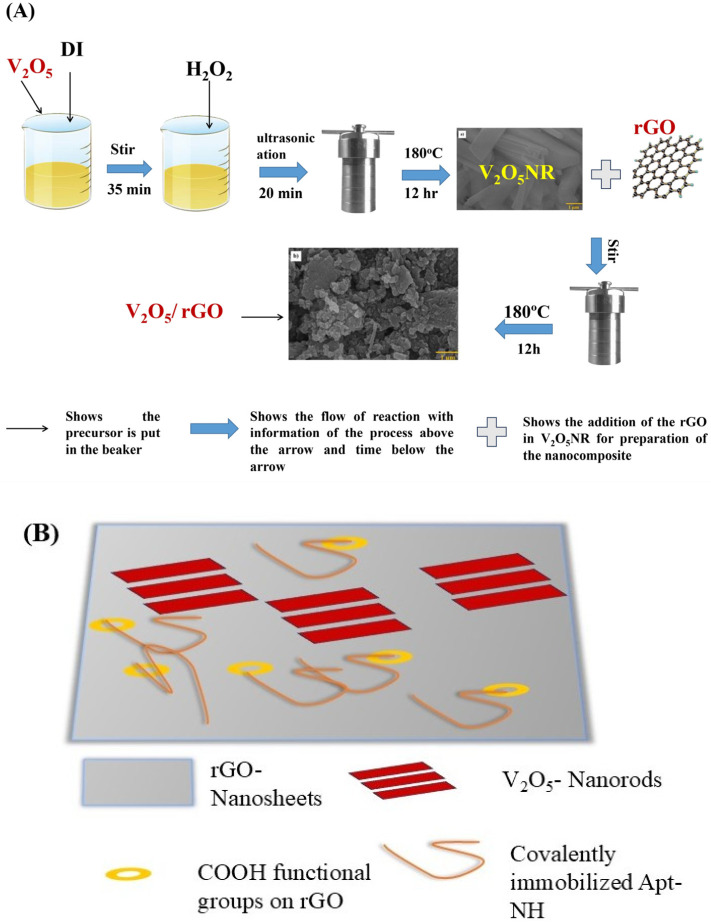
(**A**) Scheme of the synthesis of V_2_O_5_/rGO. (**B**) Illustration of the immobilization of DNA aptamers modified by amino groups (Apt-NH) on V_2_O_5_/rGO.

The V_2_O_5_/rGO composite was prepared as follows: 0.1 g of V_2_O_5_ nanorods was added to the dispersion of rGO (0.05 g in 50 mL of DI), and a homogeneous solution was obtained by magnetically stirring. Thereafter, the homogeneously stirred solution was shifted to a Teflon-lined stainless-steel container with an autoclave, which was placed in an electric oven set to 180 °C for 12 h. Finally, the V_2_O_5_/rGO was obtained by filtering the solution, as described in the section on the synthesis of V_2_O_5_ nanorods. Scheme 1A depicts the synthesis process.

### 2.3. Preparation of V_2_O_5_/rGO-Modified GCE and Immobilization of DNA Aptamers

In the first step of the sensor preparation, it is essential to polish the bare GCE with alumina powders of 0.3 µm and 0.05 µm, respectively, on a polishing pad to obtain a smooth and clean GCE surface. Afterwards, to eliminate the micro-adsorbate, the processed electrode surface was washed with DI, then immersed in ethanol, and sonicated for 5 min. Then, it was connected to the electrochemical cell containing 0.5 M H_2_SO_4_. The GCE was then electrochemically cleaned by cycling the potential in the range of 0.1–0.7 V vs. an Ag/AgCl reference electrode at a scan rate of 0.1 V/s for two sweeps. Afterwards, the electrode was cleaned extensively with DI and dried under nitrogen flow.

The surface modification was performed using the drop cast method. Briefly, a 1:0.1 ratio of V_2_O_5_/rGO and polyvinylidene fluoride (PVDF) was added to 1 mL of N-methylpyrrolidone (NMP), and a homogenous slurry was obtained by ultrasonicating for 5 min. The 2 µL of the solution was then pipetted onto the GCE surface. Finally, the electrode was left to dry in a vacuum desiccator for 24 h.

The 100 µM stock solution of Apt-NH was prepared by adding an appropriate amount of nuclease-free water, and then a 1 µM solution of Apt-NH was diluted by Tris-HCl buffer in an Eppendorf tube. Thereafter, the V_2_O_5_/rGO-modified electrode was incubated in 20 mM EDC and 50 mM NHS for 2 h and raised. Subsequentially, the electrode was incubated in the prepared 1 µM Apt-NH for 1 h and then incubated in 0.1 M ethanolamine for 30 min. An illustration of the immobilization of Apt-NH onto the V_2_O_5_/rGO sensing surface is displayed in Scheme 1B.

### 2.4. The Methods of Nanocomposite Characterization

To verify the crystallographic phase of the products, the X-ray diffractometer (XRD) Bruker D8 Advance (Bruker, Bremen, Germany) was used. The radiation source was a monochromatic CuK (λ = 1.54 Å), working at a current of 40 mA and a voltage of 40 kV, covering a range of 2θ from 5° to 80°, with a step of 0.02°. The TESCAN MIRA3 LMH (Brno, Czech Republic) was used to image the sensing surface using field emission scanning electron microscopy (FE-SEM) at 30 kV. The elemental composition was analyzed by energy-dispersive X-ray spectroscopy (EDX) mapping using the TESCAN MIRA3 LMH (Brno, Czech Republic). The pore diameter and the specific surface area of the as-prepared materials were measured by BJH and BET techniques, respectively, utilizing the ForS-BET3 (MPore 3, CAD instruments, Naucelle, France). To examine the impact of rGO on the structural attributes of the nanocomposite, Raman spectroscopy was used (Xplora plus, Horiba Scientific, Palaiseau, France). To analyze the functional groups and chemical bonds present in the materials, Fourier transform infrared (FTIR) spectra were recorded by employing the Alpha ECO—ATR (Bruker, Bremen, Germany) in the ATR mode. Electrochemical studies using cyclic voltammetry (CV) and differential pulse voltammetry (DPV) were performed using the µAutolab III electrochemical workstation (Eco Chemie, Amsterdam, The Netherlands).

### 2.5. The Electrochemical Detection of Hg(II)

In this study, we used a three-electrode system working at room temperature (RT) and in ambient conditions. In the system, the Ag/AgCl electrode was used as the reference electrode, while the counter electrode was platinum, and the working electrode was a modified GCE with a diameter of 2 mm (the electrodes were purchased from CH Instruments Inc. (Austin, TX, USA)). A CV with the potential range of 0.1–0.7 V and a typical scan rate of 0.1 V/s was used for the characterization of the modified electrodes in Tris-HCl containing 5 mM Fe(CN)_6_^3−/4−^, pH 7.4, and the DPV was applied for the detection of HMIs in 0.2 M acetate buffer, pH 5.

## 3. Results and Discussion

### 3.1. The Structural and Elemental Study of Nanocomposites

**Figure 1:**
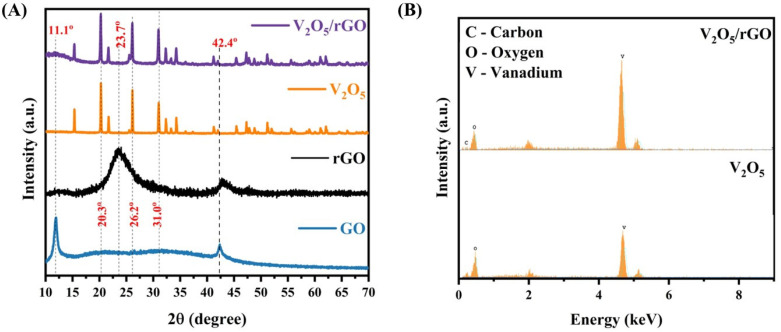
(**A**) XRD pattern of V_2_O_5_/rGO, V_2_O_5_, rGO, and GO. (**B**) EDX pattern of V_2_O_5_/rGO and V_2_O_5_.

The XRD patterns of the as-prepared samples were captured for a structural analysis and a phase study. As depicted in Figure 1A, the V_2_O_5_ nanorods demonstrate a polycrystalline structure, and, according to the diffraction pattern, the orthogonal symmetry phase of V_2_O_5_ can be confirmed, which is as per the JCPDS card no. #72–0433 [34]. The prominent peaks are recorded at the 2θ locations 20.3°, 26.2°, and 31.0°, which are associated with the (010), (101), and (310) planes, respectively. Furthermore, the observation concluded that the crystal structure of V_2_O_5_ belongs to the primitive monoclinic notation miller Pmnm (59) space group, having the following lattice parameters: a = 11.51 Å, b = 4.369 Å, c = 3.563 Å. Additionally, sharp and intense diffraction peaks suggest the excellent crystallite, calculated as 85.8%, with no additional peaks of a secondary phase observed, which concludes the high purity of the crystal phase. Additionally, the crystal size was calculated by the Debye–Scherrer equation, and it was 746.5 nm. As observed in the diffraction pattern of GO, the peak at 2θ = 11.1° indicates the presence of an oxygen functional group within the graphene sheets, which affirms the synthesis of GO (001). The additional peak at the 2θ location 42.4° appears in the pattern resulting from the presence of SP^2^ graphite in the sample. Following the thermal treatment, the peak observed at 2θ = 11.1° disappeared entirely, indicating a substantial reduction in GO. Additionally, a new broad peak at 2θ = 23.7° emerged, corresponding to rGO (002) [33].

EDX mapping was employed for the analysis of elements present in the samples, and the recorded EDX pattern is depicted in Figure 1B. From the elemental map of V_2_O_5_, it is seen that the significant peaks correspond to the V and O elements, and, in the EDX pattern of V_2_O_5_/rGO, an extra-prominent peak C element was observed. Moreover, no other prominent peaks, other than V, O, C, and the material used for the coating, were observed, substantiating the high purity of the prepared sample, which is consistent with the XRD observation.

### 3.2. The Morphology Study and BET Analysis

**Figure 2:**
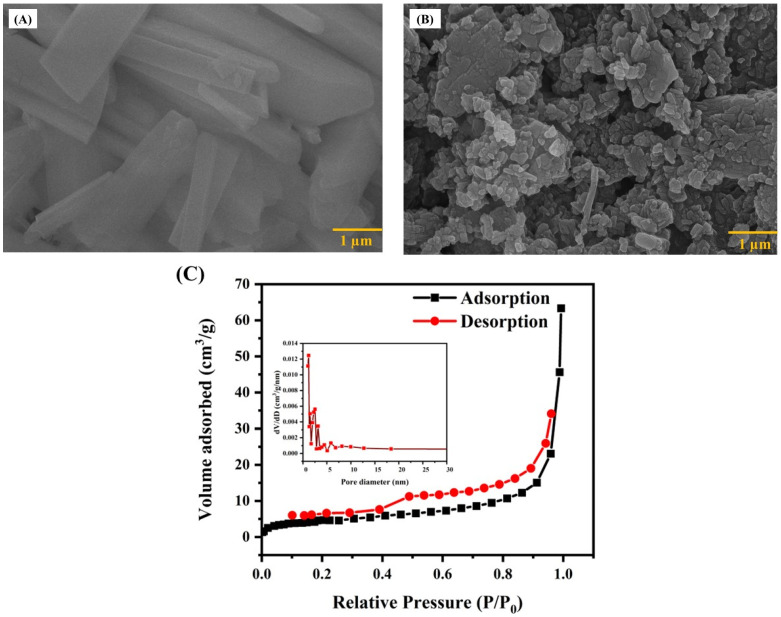
FE-SEM image of (**A**) V_2_O_5_ nanorod, (**B**) V_2_O_5_/rGO nanocomposite, and (**C**) N_2_ adsorption–desorption isotherms of V_2_O_5_/rGO.

The surface structures of pristine V_2_O_5_ and the V_2_O_5_/rGO nanocomposite were examined using FE-SEM. The images acquired are presented in Figure 2A,B. From the FE-SEM image (Figure 2A), it was noted that V_2_O_5_ exhibits a rod-like structure with an average dimension length of 1.35 µm and a width of 90 nm. Also, Figure 2B illustrates that the V_2_O_5_ nanorods have symmetrically adhered to the rGO nanosheets, which is in good concordance with the XRD results. Moreover, it can be observed that the V_2_O_5_/rGO nanocomposite possesses better porosity than the pristine V_2_O_5_ because of the advantage in the structural design of rGO [35]. The porosity of the nanocomposite plays a vital role in the enhanced sensing performance [36].

The surface properties of the material, including the pore distribution, specific surface area, and porosity, were examined utilizing the Brunauer–Emmett–Teller (BET) method, as depicted in Figure 2C. Furthermore, the porous structure is suggested by the type-IV isotherm profile with a hysteresis loop observed in the V_2_O_2_/rGO composite, indicating a mesoporous structure. It means that V_2_O_5_/rGO exhibits a structure with either regularly arranged pores or spaces between particles, which is beneficial for the adsorption of HMIs [37]. Based on these data, it has been estimated that the V_2_O_5_/rGO nanocomposite exhibits specific surface areas of 30.52 m^2^/g and an average Barrett–Joyner–Halenda (BJH) pore size of 12.14 nm.

### 3.3. The Spectroscopy Study of the Sensing Material

**Figure 3:**
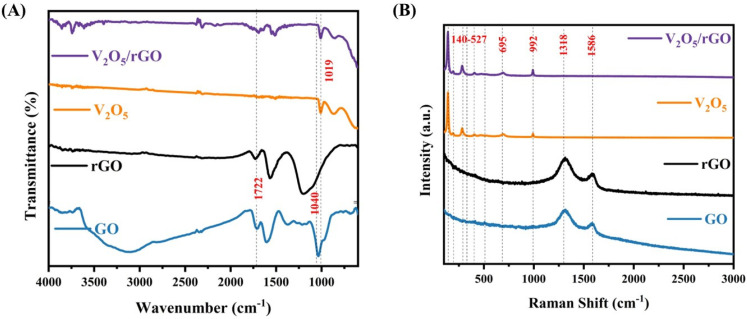
(**A**) FTIR spectrum, (**B**) Raman spectrum of V_2_O_5_/rGO, V_2_O_5_, rGO, and GO.

After the structural and morphological confirmations, the samples were analyzed by the spectroscopic tools viz. FTIR and Raman spectroscopy. Firstly, FTIR spectra were recorded to validate the potential electronic interactions and the modifications in the chemical properties of the sensing materials. These spectra are presented in Figure 3A. The rGO spectrum exhibits peaks at 1040 cm^−1^ and 1722 cm^−1^, indicating C=O stretching vibrations, with a slight downward shift, as compared to the peak present in the GO spectra, confirming the reduction of GO to rGO and the presence of oxygen-containing groups on its surface [38]. Moreover, there are oxygen configurations in the structure, including the vibrational modes of carboxyl (COOH) (1650–1750 cm^−1^) [39]. The band at 1019 cm^−1^ present in the V_2_O_5_ spectra exhibited a stretching vibration of the terminal V=O (vanadyl), associated with the preparation of V_2_O_5_ [40]. Also, an extra peak around 1722 cm^−1^ in the V_2_O_5_/rGO nanocomposite is ascribed to the presence of rGO in the sample.

Thereafter, to analyze the molecular structure, Raman spectra of the sample were acquired, as illustrated in Figure 3B. Raman shifts identified at 140 cm^−1^, 193 cm^−1^, 283 cm^−1^, 304 cm^−1^, 407 cm^−1^, 480 cm^−1^, 527 cm^−1^, 695 cm^−1^, and 992 cm^−1^ in the spectra of V_2_O_5_ corresponded to the successful synthesis of V_2_O_5_ [34]. The peaks observed at 140 cm^−1^ and 193 cm^−1^ particularly correspond to external VO_5_—VO_5_ modes, and peaks identified at 283 cm^−1^ and 407 cm^−1^ were related to V=O bending vibration. Moreover, peaks located at 304 cm^−1^ and 695 cm^−1^ were observed due to a triply coordinated oxygen (V_3_—O) stretching mode, and spectra located at 480 cm^−1^ and 527 cm^−1^ were aroused due to bending vibration of the V—O—V bond. The stretching modes of the V=O bond were confirmed from the band observed at 992 cm^−1^, which is in accordance with FTIR.

The prominent Raman bands observed at 1318 cm^−1^ and 1586 cm^−1^ in the GO spectra correspond to the characteristic D and G bands. This typically indicates that the out-of-plane vibrations result from structural defects, and the in-plane vibrations result from sp^2^-bonded carbon atoms, which is also confirmed by XRD. However, the D and G bands were shifted to locations 1313 cm^−1^ and 1582 cm^−1^ in the rGO. In the spectra of V_2_O_5_/rGO nanocomposite, the D and G bands were not identified because of their very low intensity, as compared to the intensity of V_2_O_5_ peaks, which confirms the successful preparation of the samples. In addition, the I_D_/I_G_ ratio of GO was calculated as 1.32, while for rGO, it increased to 1.59, which is typical for the thermal reduction method, as the oxygen functional groups in the GO were reduced [41]. Furthermore, the I_D_/I_G_ ratio of the composite (1.62) was higher than that of rGO (1.59), indicating the emergence of defects on the composite surface and an increased disorder. These surface defects play a crucial role in enhancing the specific surface area, which is vital for sensing applications.

### 3.4. The Electrochemical Characterization of Nanocomposite Material

**Figure 4:**
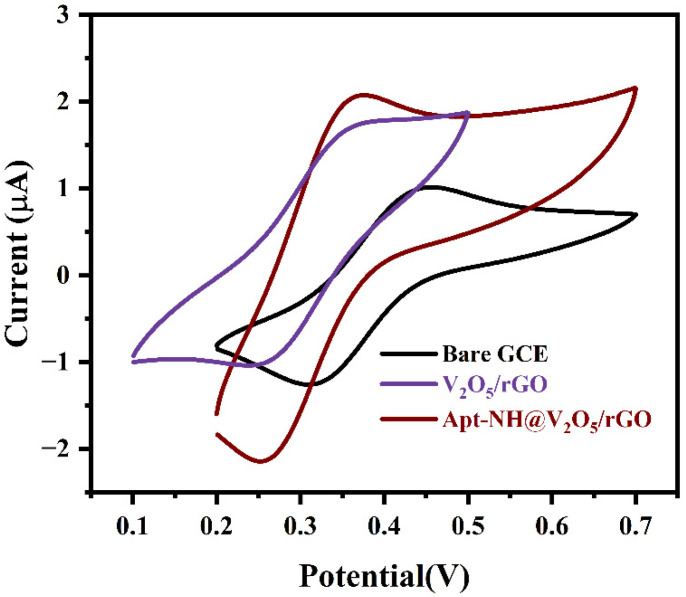
Cyclic voltammograms of bare GCE, V_2_O_5_/rGO, and Apt-NH@V_2_O_5_/rGO.

The cyclic voltammetry (CV) was used to analyze the electrochemical properties of the bare GCE, V_2_O_5_/rGO, and Apt-NH@V_2_O_5_/rGO-modified GCE in 5 mM ferri-ferrocyanide. Figure 4 presents the CV of the electrodes. It was observed from the voltammograms that bare GCE shows very poor electrochemical activity, but, after the modification by the V_2_O_5_/rGO nanocomposite, there was a remarkable increase in anodic and cathodic current, showing the excellent electrochemical activity. Only a significant anodic peak was noted at 0.36 V and a cathodic peak at 0.25 V, which correspond to the V_2_O_5_/rGO redox couple. Moreover, after the immobilization of Apt-NH on the V_2_O_5_/rGO-modified GCE, there was a substantial increase in the anodic and cathodic current. The voltammogram of Apt-NH@V_2_O_5_/rGO indicates that there is no apparent shift in the anodic and cathodic peaks. This suggests that the covalent immobilization of Apt-NH does not significantly disturb the electrical properties of the nanocomposite. Furthermore, the improved electrochemical activity of Apt-NH@V_2_O_5_/rGO over the V_2_O_5_/rGO-modified electrode proves the excellent electrochemical sensing performance.

### 3.5. Detection of Hg(II)

**Figure 5:**
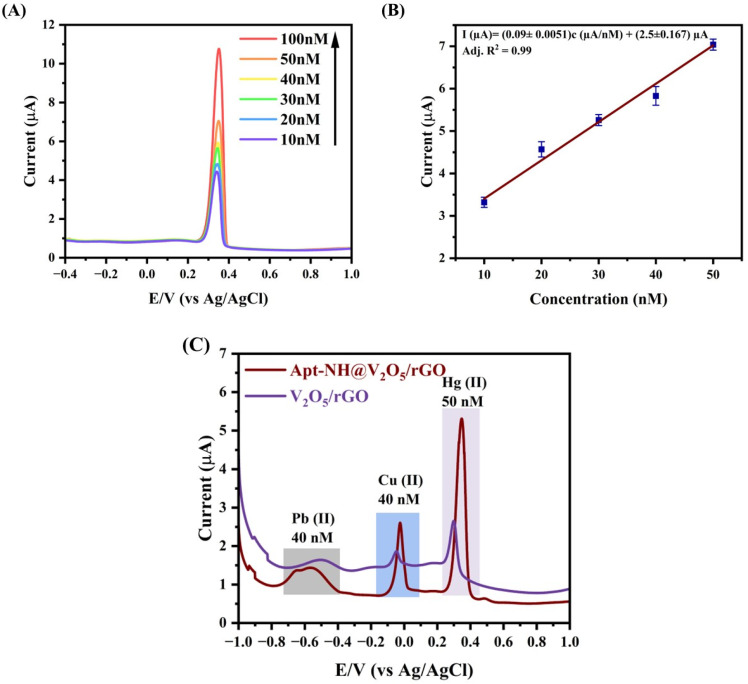
(**A**) DPV response curves for Hg(II) ions ranging from 10 to 100 nM of Apt-NH@V_2_O_5_/rGO. Arrow indicates increased concentration of Hg(II). (**B**) Calibration plot for Hg(II) concentrations. The results are mean ± SD, obtained from 5 independent experiments. The blue squares are the experimental results, and the red line is the linear regression fit. (**C**) Interference study for naked V_2_O_5_/rGO surface (blue) and those with immobilized aptamers (red) in the presence of 40 nM of Hg(II), Cu(II), and Pb(II).

The Apt-NH@V_2_O_5_/rGO-modified GCE was exposed to various concentrations of Hg(II) ions in a range from 10 nM to 100 nM. The detection of Hg(II) was performed by DPV in 0.2 M acetate buffer (pH 5). The resulting voltammogram is presented in Figure 5A. It is evident that the current peak increases gradually with the increasing Hg(II) concentration. This was confirmed by a plot of the current vs. Hg(II) concentrations (Figure 5B). The peak current linearly increased with the increasing Hg(II) concentration. The semiconducting nature, high surface area, and mesoporous structure can enhance the chemical reactions involving electron transfer at the electrode surface in the presence of Hg(II). Therefore, as the concentration of Hg(II) increases in the electrolyte, a rise in the electron transfer at the sensor surface occurs, which is reflected in the increase in the DPV peak current [42]. Good linearity was confirmed by a correlation coefficient R^2^ = 0.99. Using these data, we determined the limit of detection (LOD) based on the following equation: LOD = 3σ/b, where σ is the standard deviation of the intercept at the current axis, and b is the slope of the curve [43]. We obtained an LOD of 5.57 nM, which is under the maximum permissible level of Hg(II) ions of 6 ppb (30 nM) permitted by the WHO for river water [32]. The obtained results reveal that Apt-NH@V_2_O_5_/rGO has excellent binding efficacy toward the detection of Hg(II) ions. This shows the potential ability of the developed sensor for practical sample analyses.

Detecting Hg(II) ions selectively in solutions is challenging due to the occurrence of other metal ions in real samples. In this study, we, therefore, analyzed the selectivity of the detection in the presence of potentially interfering HMIs (viz. Pb(II), Cu(II), Zn(II), Cd(II), Fe(II), and Co(II)). Figure 5C shows the DVP of the Apt-NH@V_2_O_5_/rGO-modified GCE and V_2_O_5_/rGO-modified GCE for 40 nM Hg(II) in the presence of 40 nM of the other HMIs mentioned above. It can be seen that voltametric peaks are observed only for Pb(II), Cu(II), and Hg(II), but the peaks for Pb(II) and Cu(II) were much lower in comparison with the peak for Hg(II). Moreover, the peaks for Pb(II) and Cd(II) were shifted toward more negative potential values. For the other ions mentioned above, no significant peaks were observed. This suggests that other HMIs do not interfere with the Hg(II) ions, which is evidence of the good selectivity of the developed sensor. Moreover, the voltametric peak was significantly enhanced after the immobilization of Apt-NH on the electrode surface compared to that of the V_2_O_5_/rGO-modified GCE. This can be due to the capturing of Hg(II) by aptamers, which facilitates their approach to the nanocomposite surface. The resulting increased Hg(II) concentration caused a rise in the current amplitude. This shows the potential ability of the Apt-NH@V_2_O_5_/rGO-modified GCE for the selective and sensitive detection of Hg(II).

### 3.6. Repeatability and Stability of the Aptasensor

**Figure 6:**
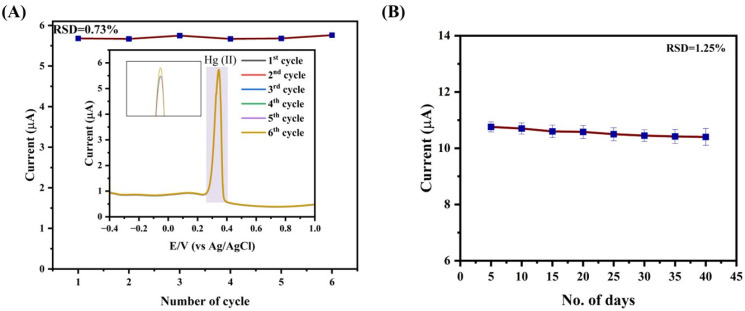
(**A**) Repeatability of Apt-NH@V_2_O_5_/rGO for the detection of Hg(II) ions. (**B**) Stability of the Apt-NH@V_2_O_5_/rGO sensor. Results are mean ± SD obtained from 5 independent experiments. Blue squares are the experimental results, and red line connecting the experimental points.

Repeatable experiments were carried out for the detection of Hg(II) to evaluate the reliability of the Apt-NH@V_2_O_5_/rGO-modified GCE sensor. The corresponding results are depicted in Figure 6A. This was estimated by recording the DPV voltammogram at the same conditions and using the same electrolyte (40 nM concentration of Hg(II) in a 0.2 M acetate buffer, pH 5) at the same Apt-NH@V_2_O_5_/rGO-modified GCE over six cycles, presented in the inset of Figure 6A. There were no apparent changes in the DPV response of Hg(II) with its ongoing detection. The high accuracy of the sensor can be illustrated by its relative standard deviation value (RSD), which was calculated to be 0.73% based on six cycles of the current response. Furthermore, to investigate the stability of the Apt-NH@V_2_O_5_/rGO sensor, the DPV response was recorded for 40 days with intervals of five days, while the electrolyte remained the same at a concentration of 100 nM Hg(II) in a 0.2 M acetate buffer, pH 5. As illustrated in Figure 6B, the current response of the sensor remains practically constant over 40 days, with an excellent RSD of 1.25%. The results reveal that the Apt-NH@V_2_O_5_/rGO-based sensor demonstrates excellent repeatability and stability, which is crucial for practical applications. A comparative performance of the Apt-NH@V_2_O_5_/rGO-modified GCE with other work on Hg(II) detection (Table 1) shows that the developed aptasensor has sufficient sensitivity and stability, allowing for detection with an LOD below the MPL.

As can be seen from Table 1, various sensing strategies for the electrochemical detection of Hg(II) ions differ by the application of various nanomaterials and their composites, the different aptamer designs (guanine quadruplexes, hairpin conformation), and the various electrochemical methods of detection. A rather high variation in the applications of nanocomposite materials shows different sensor sensitivity. However, even the relatively simple nanocomposites used, for example, in our work allow for an efficient and sufficiently sensitive Hg^2+^ detection. The application of the electrochemical method used depends on the sensing strategy. For example, for the detection of Hg^2+^ forming T − Hg^2+^ − T bridges, the DPV is rather effective. This method is most often used in the electrochemical detection of HMIs. But for the electrochemical characterization of the sensing layer, electrochemical impedance spectroscopy (EIS) is rather useful. Except for nanocomposites, even interdigitated gold layer-based field effect transistors (FETs) are very effective for HMI detection [21], although the preparation of such sensors needs special methods. At the same time, the advantage of gold layers consists of a relatively simple immobilization of the thiolated aptamers by chemisorption. The aptamer design plays an important role in the detection of HMIs, as has been demonstrated in a recent paper by Zeng et al. [31]. The optimization of the aptamer hairpin structure resulted in an increased sensitivity and extension of the linear range of Hg^2+^ detection. An important approach also consists of the application of special peptides for surface modification, providing sensor antifouling properties [46]. The progress in the application of nanomaterials with immobilized aptamers for the detection of Hg(II) has been discussed in a comprehensive review by Raina et al. [54].

## 4. Conclusions

In this paper, hydrothermal synthesis of V_2_O_5_/rGO was performed, and the resulting nanocomposite was used for the preparation of an aptamer-based sensor for the electrochemical detection of Hg(II). The V_2_O_5_/rGO nanocomposite was drop casted on the surface of GCE, onto which DNA aptamers were covalently attached. The aptasensor demonstrated excellent sensitivity and selectivity of Hg(II) detection, with an LOD of 5.57 nM and a linear range of 10–50 nM. Furthermore, the sensors showed outstanding repeatability and stability over 40 days toward the detection of Hg(II), with calculated RDS values of 0.73% and 1.25%, respectively. The proposed aptasensor has good potential for the analysis of water pollution by Hg(II). The sensor can be applicable for the detection of Hg(II) in water samples, such as drinking or river water, that do not require special pretreatment, except for microfiltration for the removal of mechanical impurities. However, the detection of mercury in food samples needs special attention, including the preparation of sensing surfaces with antifouling properties. From the literature overview, it can also be mentioned that T − Hg^2+^ − T bridges are formed in various buffers, such as acetate, PBS, or Tris-HCl. This is evidence of the high stability and versatility of these bridges. This was also approved in our long-time experiments that revealed aptasensor stability for at least 40 days.

## Figures and Tables

**Table 1:** Comparison of the electrochemical detection of Hg(II) using DNA aptamer-based sensors.

Electrode Material	Linear Range	LOD	References
Ps-AuNP-decorated ssDNA	2.5–100 nM	24.9 nM	[27]
rGO-AuNPs	17 nM–2.8 µM	6 nM	[28]
CeO_2_-Apt/GCE	Log (10 pM–100 µM)	0.35 pM	[29]
PLL/BP-PG	Log (1 nM–10 µM)	45 pM	[30]
Au surface of FET	Log (10 pM–10 µM)	4.68 pM	[31]
GCE/poly(CoTABImPc)	10–400 nM	3 nM	[44]
Rotating Au disk electrode	33–233 nM	1.92 nM	[45]
Gold nanostars	1–500 nM	2.5 nM	[46]
Screen-printed gold electrode	25 nM–1.5 µM	6.5 nM	[47]
MCH/HBA/PBA/Au *	Log (0.1–100 nM)	19 pM	[48]
AuNRs/SPE	1 pM to 1 μM	0.3 pM	[49]
3D porous AuNPs/MXene	10 fM–0.1 nM	2.69 fM	[50]
Apt-pep-Au/Ag/PEDOT/GCE	Log (0.01–100 nM)	2.3 pM	[51]
Ag@Co-Succinate-MOF	0.7–10 nM	0.3 nM	[52]
Co-MOF@T-COOH	1–60 nM	3.69 nM	[53]
Apt-NH@V_2_O_5_/rGO	10–50 nM	5.57 nM	This work

* Dual aptasensor for the simultaneous detection of Pb^2+^ (LOD 0.98 pM) and Hg^2+^ (LOD 19 pM). Apt-pep-Au: aptamer peptide complexes chemisorbed on gold; AuNPs: gold nanoparticles; AuNRs: gold nanorods; BP-PG: black-phosphorus porous graphene; Co-Succinate: cobalt–succinate complex; Co-MOF: cobalt metal-organic framework; Cu^2+^-Mel: Cu^2+^-melamine; FET: field effect transistor; HBA: Hg^2+^ binding aptamer; GCE: glassy carbon electrode; MCH: 6-mercapto-1-hexanol; MXene: a class of 2D inorganic compounds containing a metal (M); PBA: Pb^2+^ binding aptamer; PEDOT: poly-3,4-ethylendioxythiophen; PLL: polylysine; poly(CoTABImPc): polymerized cobalt(II) tetracarboxylic acid phthalocyanine; RGO: reduced graphene oxide; SPE: screen-printed electrode; T-COOH: thymine-1-acetic acid.

## Data Availability

Data are available upon request.
